# Diffusion Basis Spectrum Imaging Detects Axonal Loss After Transient Dexamethasone Treatment in Optic Neuritis Mice

**DOI:** 10.3389/fnins.2020.592063

**Published:** 2021-01-22

**Authors:** Tsen-Hsuan Lin, Jie Zhan, Chunyu Song, Michael Wallendorf, Peng Sun, Xuan Niu, Ruimeng Yang, Anne H. Cross, Sheng-Kwei Song

**Affiliations:** ^1^Department of Radiology, Washington University School of Medicine, St. Louis, MO, United States; ^2^Department of Biomedical Engineering, Washington University in St. Louis, St. Louis, MO, United States; ^3^Division of Biostatistics, Washington University School of Medicine, St. Louis, MO, United States; ^4^Department of Neurology, Washington University School of Medicine, St. Louis, MO, United States; ^5^Hope Center for Neurological Disorders, Washington University School of Medicine, St. Louis, MO, United States; ^6^Department of Radiology, Guangzhou First People’s Hospital, School of Medicine, South China University of Technology, Guangzhou, China; ^7^Department of Radiology, The First Affiliated Hospital, Nanchang University, Jiangxi, China

**Keywords:** axonal loss, optic neuritis (ON), multiple sclerosis (MS), diffusion MRI, dexamethasone, anti-inflammation, Diffusion basis spectrum imaging (DBSI)

## Abstract

Optic neuritis is a frequent first symptom of multiple sclerosis (MS) for which corticosteroids are a widely employed treatment option. The Optic Neuritis Treatment Trial (ONTT) reported that corticosteroid treatment does not improve long-term visual acuity, although the evolution of underlying pathologies is unclear. In this study, we employed non-invasive diffusion basis spectrum imaging (DBSI)-derived fiber volume to quantify 11% axonal loss 2 months after corticosteroid treatment (vs. baseline) in experimental autoimmune encephalomyelitis mouse optic nerves affected by optic neuritis. Longitudinal DBSI was performed at baseline (before immunization), after a 2-week corticosteroid treatment period, and 1 and 2 months after treatment, followed by histological validation of neuropathology. Pathological metrics employed to assess the optic nerve revealed axonal protection and anti-inflammatory effects of dexamethasone treatment that were transient. Two months after treatment, axonal injury and loss were indistinguishable between PBS- and dexamethasone-treated optic nerves, similar to results of the human ONTT. Our findings in mice further support that corticosteroid treatment alone is not sufficient to prevent eventual axonal loss in ON, and strongly support the potential of DBSI as an *in vivo* imaging outcome measure to assess optic nerve pathology.

## Introduction

Multiple sclerosis (MS) is an inflammatory demyelinating disease affecting the central nervous system (CNS), including brain, optic nerves, and spinal cord. Anti-inflammation treatment using corticosteroids is often used to suppress relapses. Corticosteroids are thought to shorten duration of MS relapses but not to alter the long-term outcome. Optic neuritis (ON) occurs frequently, often as the initial episode, in MS (Michalski et al., 1981; Gonzalez-Hernandez et al., 2015). Corticosteroids are widely used to treat ON in MS patients (Beck et al., 1993; Bennett et al., 2015) and are also effective in reducing clinical signs of murine experimental autoimmune encephalomyelitis (EAE) (Dustman and Snyder, 1981), an animal model of MS. Corticosteroids have multiple effects, including anti-inflammatory and immunosuppressive effects, reduction of blood–brain barrier (BBB) permeability and alteration of ion channel activity (Levitan et al., 1991; Boumpas et al., 1993; Wust et al., 2008; Myhr and Mellgren, 2009; Coutinho and Chapman, 2011). The seminal Optic Neuritis Treatment Trial (ONTT) reported no long-term functional benefits from either intravenous or oral corticosteroid treatment of acute ON, but did find expedited recovery of visual function (Valberg et al., 1981; Gal et al., 2015). Known adverse effects of corticosteroids in humans are many, including reduced glucose metabolism, cataract formation, joint injury and loss of bone density. Experimentally, several reports have also shown neuronal cell loss in animal models (Diem et al., 2003; Lieberman et al., 2011; Muller et al., 2014). Hence, we have taken a longitudinal and non-invasive imaging assessment of the evolution of optic nerve pathology in murine ON, culminating in histological assessment, to improve the understanding of the impact of corticosteroid treatment.

Magnetic resonance imaging (MRI) plays a vital role in diagnosing and assessing disease progression in people with MS. For instance, contrast-enhanced lesions and chronic T1-weighted hypointensities reflect inflammation, and axonal loss, respectively, but are at best only semi-quantitative (Ge, 2006; Ceccarelli et al., 2012; Llado et al., 2012). Axonal loss is a critical mechanism of irreversible neurological disability (Kornek et al., 2000; Wujek et al., 2002; Medana and Esiri, 2003). A non-invasive biomarker to quantify the extent of axonal loss and residual axon injury would greatly improve the understanding of evolution of injury and help stratify therapies for individual MS. Magnetization transfer ratio (MTR) and *N*-acetyl aspartate to creatine ratio determined by magnetic resonance spectroscopy (MRS) are usually considered the best imaging biomarkers for myelin and axon integrity, respectively, in people with MS (Davie et al., 1999; Bjartmar et al., 2000; Schmierer et al., 2004). Diffusion-tensor-imaging (DTI)-derived axial diffusivity (AD, also denoted as λ_∥_) and radial diffusivity (RD, also denoted as λ_⊥_) have been used to more specifically assess axonal injury, and demyelination. However, the DTI model is confounded by coexisting pathologies such as inflammation and axon loss on AD and RD (Wang et al., 2011b; Chiang et al., 2014). Therefore, we developed diffusion basis spectrum imaging (DBSI) to analyze diffusion-weighted images obtained with multi-direction and multi-b-value schemes. DBSI more accurately detects and quantifies co-existing white-matter pathologies in EAE-affected mice and people with MS (Wang et al., 2007, 2011a, 2014, 2015; Chiang et al., 2014).

Optic neuritis frequently occurs in murine EAE, as seen in people with MS. In the current study, we performed longitudinal DBSI to assess injury progression in the optic nerves of EAE-affected mice undergoing treatment with a widely used corticosteroid, dexamethasone (Dex) (Wust et al., 2008; Coutinho and Chapman, 2011) followed by post-MRI immunohistochemical validation. The study was set up to reflect a typical scenario for human ON, with treatment of individual mice beginning only after signs of reduced visual acuity (VA) and stopping after 2 weeks.

## Materials and Methods

All experimental procedures involving animals were approved by Washington University Institutional Animal Care and Use Committee (IACUC) and conformed to the NIH Policy on Responsibility for Care and Use of Animals.

### Experimental Autoimmune Encephalomyelitis (EAE) Mouse Model

Fifteen 7-week-old, female C57BL/6 mice were obtained from Jackson Laboratory (Bar Harbor, ME, United States). Before immunization, mice were housed with 12-h dark/light cycle for 2 weeks. EAE was induced by active immunization with 50 μg MOG_35__–__55_ peptide emulsified (1:1) in incomplete Freund’s adjuvant (IFA) and Mycobacterium tuberculosis. Pertussis toxin (300 ng; PTX, List Laboratories, Campbell, CA, United States) was injected intravenously on the day of MOG_35__–__55_ immunization and 2 days later (Wang et al., 2007).

### Visual Acuity (VA)

Mouse VA was assessed using the Virtual Optometry System (OptoMotry, Cerebral Mechanics, Inc., Canada). Briefly, the virtual rotating columns were projected on the LCD monitors with different spatial frequencies in cycles/degree (c/d). The mouse head movement in response to the virtual column rotations was noted. The spatial frequency was starting from 0.1 c/d with step size of 0.05 c/d until the mouse stopped responding. The VA was defined as the highest spatial frequency to which the mouse responded to the virtual rotating columns. Each mouse was confirmed to have normal VA before immunization. After immunization, daily VA was assessed until VA ≤ 0.25 c/d, our definition for the onset of ON in MOG_35__–__55_ EAE mice (Chiang et al., 2014; Lin et al., 2014a,b). The first day of VA ≤ 0.25 c/d both Dex- and PBS-treated group was 13.4 ± 3.7 days post immunization. Upon Dex treatment commencement, VA was performed twice a week and 1 day before MRI scans.

### Dexamethasone Administration

When VA ≤ 0.25 c/d, ON-affected mice underwent daily intraperitoneal injection of Dex (3 mg/kg, Sigma Inc., MO, United States) or PBS for 2 weeks. The first day of VA ≤ 0.25 c/d in both Dex and PBS groups was 13.4 ± 3.7 days post immunization. Mice were alternately assigned to receive PBS or Dex until the 9th pair. The last EAE mouse was assigned to PBS group. Daily clinical scores were assessed using a standard 0–5 scoring system (Wang et al., 2014).

### Diffusion-Weighted MRI Data Acquisition

**FIGURE 1:**
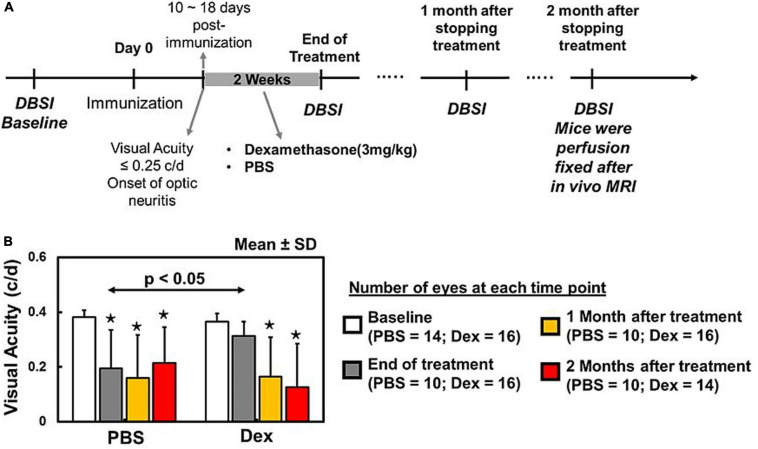
Baseline DBSI prior to active immunization of EAE mice **(A)**. Daily intraperitoneal PBS or dexamethasone injection started at the onset of optic neuritis (i.e., visual acuity (VA) < 0.25 c/d) and continued for 2 weeks. DBSI was performed at the end of the 2-week treatment, with 1- and 2-month follow-ups after the initial scan **(A)**. Dexamethasone-treated eyes exhibited baseline visual acuity (VA) that was significantly higher than PBS-treated eyes during treatment period **(B)**. One and two months after concluding treatment, VA of PBS and dexamethasone groups were significantly lower than their baseline **(B)**. There was no difference between PBS and dexamethasone groups **(B)**. The VA data suggested that dexamethasone treatment could only retain intermittent visual function during treatment period. * indicates *p* < 0.05, comparing to baseline.

Mice were anesthetized for imaging as previously described (Lin et al., 2017). *In vivo* MRI experiments were performed on a 4.7-T Agilent DirectDrive^TM^ small-animal MRI system (Agilent Technologies, Santa Clara, CA, United States) equipped with a Magnex/Agilent HD imaging gradient coil (Magnex/Agilent, Oxford, United Kingdom) capable of pulsed-gradient strengths of up to 58 G/cm and a gradient rise time ≤ 295 μs. An actively decoupled 1.7-cm receive coil was placed on the top of the mouse head for MR signal reception. The animal holder assembly, including the receive coil was placed inside an 8-cm actively decoupled volume transmit coil. Diffusion-weighted MRI data were acquired with 25-direction diffusion weighting scheme (Batchelor et al., 2003) using a multi-echo spin-echo diffusion-weighted imaging sequence (Tu et al., 2010). The following parameters were used to acquire diffusion-weighted MRI data: TR = 1.5 s, TE = 35 ms, inter-echo delay = 20.7 ms, FOV = 22.5 × 22.5 mm^2^, matrix size = 192 × 192 (zero-filled to 384 × 384), slice thickness = 0.8 mm, 25 different *b*-values (max *b*-value = 2,200 s/mm^2^), one b = 0 s/mm^2^, Δ = 18 ms, δ = 6 ms, total scan time = 2 h 4 min (Chiang et al., 2014; Lin et al., 2017). The final target image view was perpendicular to optic nerve and obtained as previously described (Spees et al., 2013; Lin et al., 2014b). A train of two echoes was co-added to form the final MR images to increase accumulated signal-to-noise ratio. Diffusion-weighted MRI was performed four times on each mouse: 2 weeks before immunization (baseline), at the end of 2-week treatment (2 weeks after onset of ON), and 1 and 2 months after stopping treatment (chronic, no longer treated) (Figure 1A).

### Diffusion Basis Spectrum Imaging (DBSI) and Diffusion Tensor Imaging (DTI)

Data was analyzed with DBSI multi-tensor and conventional DTI single-tensor analysis packages developed in-house with MATLAB (Wang et al., 2011a, 2015). The imaging data were modeled according to Eq. 1:

Sk=∑i=1NA⁢n⁢i⁢s⁢ofi⁢e-|bk⇀k|⁢λ⊥i⁢e-|bk⇀k|⁢(λ∥i-λ⊥i)⁢cos2⁡ψi⁢k

(1) +∫abf(D)e-|bk⇀k|⁢DdD (k=1, 2, 3,…, 25).

The quantities *S*_*k*_ and $\left|\vec{b_{k}}\right|$ are the signal and *b*-value of the *k*^*th*^ diffusion gradient, *N*_*Aniso*_ is the number of anisotropic tensors (fiber tracts), Ψ_*ik*_ is the angle between the *k*^*th*^ diffusion gradient and the principal direction of the *i*^*th*^ anisotropic tensor, λ*_| | i_* and λ_⊥_*_*i*_* are the AD and RD of the *i*^*th*^ anisotropic tensor, *f*_*i*_ is the signal intensity fraction for the *i*^*th*^ anisotropic tensor, and *a* and *b* are the low and high diffusivity limits for the isotropic diffusion spectrum (reflecting cellularity and edema) *f*(*D*). For a coherent fiber bundle like the optic nerve, *N*_*Aniso*_ = 1. DBSI derived *f*_*i*_ represents the density of axons derived from retinal ganglion cells (fiber fraction) in the image voxel, after resolving intra-voxel pathological and structural complications. Based on prior work, DBSI derived λ_| |_ and λ_⊥_ reflect axon and myelin integrity respectively: ↓λ_| |_ = axonal injury and ↑λ_⊥_ = demyelination. Our previous experimental findings suggest that the restricted isotropic diffusion fraction reflecting cellularity can be derived by the summation of *f*(*D*) at 0 ≤ ADC ≤ 0.6 μm^2^/ms. The summation of the remaining *f*(*D*) at 0.6 < ADC < 3 μm^2^/ms represents “hindered” isotropic diffusion, which denotes regions of tissue loss, increased inter-axonal space, vasogenic edema and CSF. The summation of *f*(*D*) at ADC = 3 μm^2^/ms represents free water.

Regions of interest (ROI) were manually drawn with ImageJ^1^ (NIH, United States) at the center of each optic nerve on the diffusion-weighted image (the edge voxel of optic nerve were avoided), which corresponded to the diffusion gradient direction perpendicular to optic nerves, to minimize partial volume effects. ROIs were then transferred to the parametric maps to calculate the mean value for individual DBSI metrics.

### ROI for DBSI Fiber Volume

A separate ROI encompassing the whole optic nerve was drawn on cross-sectional images obtained with diffusion weighting gradient direction orthogonal to optic nerve (larger than the ROIs for other DBSI metrics). The ROI included the edge voxel of optic nerve. DBSI-derived fiber volume was calculated from the optic nerve volume (the entire ROI on DWI) multiplied by the corresponding DBSI fiber fraction. The dilution effect of inflammation and surrounding CSF is considered in the fiber volume computation in the manner.

### Immunohistochemistry (IHC) of Optic Nerves

Following the final MR examination, mice were perfused with 1% phosphate-buffered saline followed by 4% paraformaldehyde in 1% phosphate-buffered saline. The brain was excised and post-fixed for 24 h before being transferred to 1% phosphate-buffered saline for storage at 4°C. For histological analysis, mouse optic nerves were embedded in 2% agar (Blewitt et al., 1982). The agar block was then embedded in paraffin wax and 5 μm thick transverse slices were sectioned for IHC. Sections were deparaffinized, rehydrated, and then blocked using 1% bovine serum albumin (BSA, Sigma Inc., MO, United States) and 5% normal goat serum solution for 30 min at room temperature to prevent non-specific binding and to increase antibody permeability. Slides were incubated overnight at 4°C with purified anti-neurofilament marker pan axonal cocktail (1:300, SMI-312, BioLegend, United States), or purified anti-neurofilament H (NF-H), phosphorylated antibody (1:300, SMI-31; BioLegend, United States) to stain total axons or only non-injured axons, respectively. Rabbit anti-myelin basic protein (MBP) antibody (1:300, Sigma Inc., MO, United States) was used to stain myelin blobs from breakdown or intact myelin sheaths (Song et al., 2003; Costello et al., 2006b; Urolagin et al., 2012). After rinsing, goat anti-mouse IgG or goat anti-rabbit IgG conjugated Alexa 488 (1:240, Invitrogen, United States) was applied to visualize immunoreactivity of phosphorylated neurofilament and MBP. Finally, slides were covered using Vectashield Mounting Medium with 4′,6-diamidino-2-phenylindole (DAPI, Vector Laboratory, Inc., Burlingame, CA, United States) to stain cell nuclei (Costello et al., 2006b; Wang et al., 2007, 2011a). Images were acquired with a Nikon Eclipse 80i fluorescence microscope equipped with 100 × oil objective and a black-and-white CCD camera with MetaMorph software (Universal Imaging Corporation, Sunnyvale, CA, United States) for entire optic nerve with the montage function.

### Histological Data Analysis

The whole field of SMI-31, MBP, and DAPI stained images at 100× magnification was captured with the same fluorescence light intensity and exposure time for each image. All captured images were converted to 8-bit gray scale and analyzed using threshold, edge enhancement, analyze particles and gray level watershed segmentation functions in ImageJ (see text footnote 1, NIH, United States). Nucleus counts were performed by signal intensity threshold on DAPI staining (Lin et al., 2014a,b).

### Statistical Analysis

Three PBS-treated EAE mice and one Dex-treated EAE mouse died before the end of the 2-week treatment. Two PBS-treated EAE mice died before the MRI scan at 1 month after treatment. At conclusion of the study, five PBS-treated and seven Dex-treated EAE mice had survived through the final DBSI scan (2 months after treatment) and histologic analysis.

For all the boxplots, whiskers extend to the minimum/maximum and the means are marked as diamonds. VA or MRI measurements were taken on each eye at baseline, end of 2-week treatment, and at 1 and 2 months after treatment. Data were analyzed with a mixed random effects repeated measures model with side, time, treatment, and time by treatment interaction as fixed effects. Degrees of freedom were adjusted with Kenward–Rogers method. A first order auto-regressive covariance structure was used to account for repeated measures. Contrasts were estimated for change from baseline. The associations of histology data with DBSI measurements at 2 months after treatment were analyzed by mixed random effects regression with correlation calculated as the mean of Pearson correlations on left and right sides.

## Results

### Recovery of Visual Function During Dexamethasone Treatment Period

Visual acuity in Dex-treated eyes were comparable to its baseline (*p* = 0.1615, Figure 1B) and significantly improved than PBS-treated eyes at the end of 2-week treatment (*p* = 0.0242, Figure 1B). One and two months after stopping treatment, both Dex- and PBS-treated eyes were significantly lower than their baseline (*p* < 0.0001, Figure 1B) and no difference between two groups (*p* = 0.3992 and *p* = 0.3570 for 1 and 2 months, respectively).

### DBSI: Acute Anti-inflammatory Effects of Dexamethasone

**FIGURE 2:**
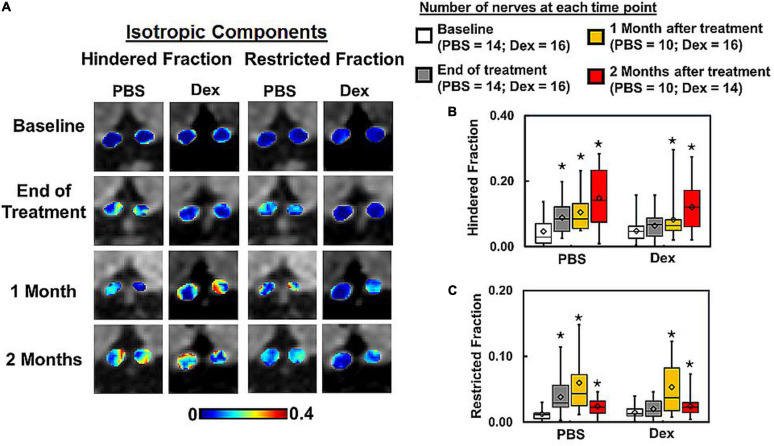
DBSI-derived hindered (putative vasogenic edema marker) and restricted (putative cellularity marker) isotropic diffusion tensor fraction maps **(A)** from the representative PBS- and dexamethasone-treated mice were compared. Dexamethasone suppressed inflammation, i.e., the putative inflammation markers did not differ from baseline values including hindered (*p* = 0.51) and restricted (*p* = 0.46) fractions, at the end of 2-week treatment **(B,C)**. In contrast, hindered (*p* < 0.05) and restricted (*p* < 0.05) fractions significantly increased (reflecting prominent inflammation) at the end of 2-week PBS treatment. At 1 and 2 months after cessation of PBS or dexamethasone treatment, hindered (*p* < 0.05) and restricted (*p* < 0.05) isotropic diffusion fractions increased compared to baseline **(A–C)**. Summary box plots show that these isotropic diffusion metrics were elevated to similar degree in both PBS- and dexamethasone-treated optic nerves at 1 and 2 months **(B,C)**. The latter suggest the ineffectiveness of dexamethasone after cessation of treatment. There was no statistically significant difference between treatment groups at each matched time point. * indicates *p* < 0.05, comparing to baseline.

Comparing to the baseline (within each treatment group), significantly increased DBSI hindered (elevated by 90% from baseline, *p* = 0.038, Figure 2B and Table 1) and restricted isotropic (increased by 285% from baseline, *p* = 0.0074, Figure 2C and Table 1) diffusion fractions were seen in optic nerves at the end of the 2-week PBS treatment (Figure 2). In contrast, moderate but not statistically significantly increased DBSI hindered (28%, *p* = 0.52, Figure 2B and Table 1) and restricted isotropic (48%, *p* = 0.46, Figure 2C and Table 1) fractions were seen 2 weeks after the Dex-treatment. The extent of increased hindered isotropic diffusion fraction (putative marker of edema, increased inter-axonal space, or tissue loss) and restricted isotropic diffusion fraction (putative marker of cellularity) was significantly increased at 2 months after Dex- (187%, *p* = 0.0002 and 174%, *p* = 0.0071 from baseline, respectively and Table 1) or PBS-treatments (147%, *p* = 0.0009 and 207%, *p* = 0.0093 from baseline, respectively and Table 1). With our limited mouse number, none of the DBSI metrics exhibited a statistically significant difference between the two treatment groups at any of the examined time points.

**TABLE 1:** Group averaged of DTI or DBSI metrics of EAE mice with PBS (*n* = 7 for baseline and end of treatment, *n* = 5 for 1 and 2 months after treatment) and dexamethasone (*n* = 8 for baseline, end of treatment, and 1 month after treatment, *n* = 7 for 2 months after treatment) treatment.

		**Baseline**	**End treatment**	**1 month**	**2 months**
DTI ADC (μm^2^/ms)	PBS	0.740.03	0.740.06	0.740.06	0.720.06
	Dexamethasone	0.730.03	0.740.07	0.710.06	0.730.04
DBSI axial diffusivity (μm^2^/ms)	PBS	1.880.15	*1.760.22	*1.800.18	*1.730.21
	Dexamethasone	1.860.11	1.860.16	*1.820.18	*1.760.10
DTI axial diffusivity (μm^2^/ms)	PBS	1.720.25	*1.490.38	*1.520.21	*1.500.24
	Dexamethasone	1.740.12	1.700.22	*1.560.18	*1.490.18
DBSI radial diffusivity (μm^2^/ms)	PBS	0.180.52	*0.260.09	*0.290.08	0.230.06
	Dexamethasone	0.190.05	0.230.05	0.230.05	*0.260.08
DTI radial diffusivity (μm^2^/ms)	PBS	0.250.16	0.350.11	0.350.10	0.330.06
	Dexamethasone	0.210.04	0.260.05	0.300.07	0.360.11
DBSI non-restricted fraction	PBS	0.050.04	*0.090.05	*0.090.05	*0.080.12
	Dexamethasone	0.040.04	0.050.04	*0.080.07	*0.120.07
DBSI restricted fraction	PBS	0.020.01	*0.060.05	*0.060.05	*0.050.05
	Dexamethasone	0.020.02	0.030.02	*0.050.04	*0.060.04
DBSI fiber signal fraction	PBS	0.790.07	*0.700.07	*0.730.07	*0.710.11
	Dexamethasone	0.790.05	0.790.04	0.750.09	*0.690.10
DWI-derived optic nerve volume (mm^3^)	PBS	0.090.01	0.110.02	0.100.01	0.090.02
	Dexamethasone	0.100.01	0.100.01	0.100.01	0.100.03
DBSI-derived fiber volume (mm^3^)	PBS	0.0730.007	0.0730.012	0.0710.014	0.0650.013
	Dexamethasone	0.0780.006	0.0750.006	0.0770.014	*0.0700.019

* Indicates *p* < 0.05, comparing to baseline.

### DBSI: Delayed Axon/Myelin Injury With Dexamethasone Administration

**FIGURE 3:**
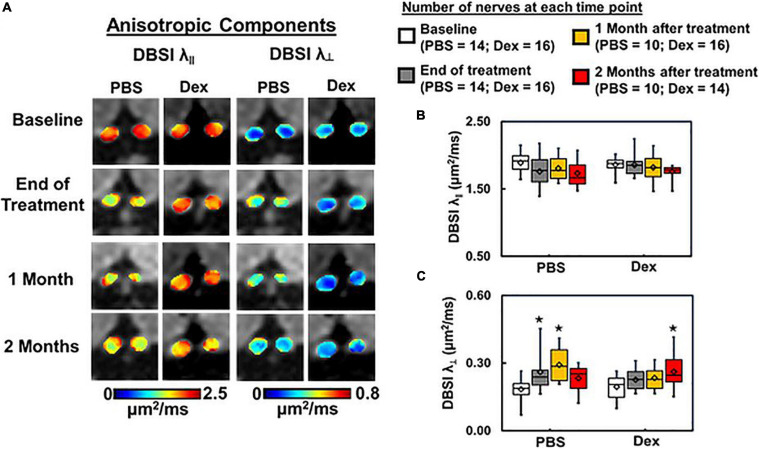
DBSI-derived axial (λ_∥_) and radial (λ_⊥_) diffusivity maps **(A)** from the same two representative mice as in Figure 2. Optic nerves from the PBS-treated mouse developed axon and myelin injury as revealed by the decreased λ_∥_ and increased λ_⊥_ **(A)**. In contrast, optic nerves from the dexamethasone treated mouse were minimally affected, as reflected by the near baseline λ_∥_ and λ_⊥_ values at the end of 2-week treatment **(A)**. Box plots of DBSI-derived λ_∥_ **(B)** and λ_⊥_ **(C)** from the two cohorts of mice revealed that at the end of the 2-week treatment period, DBSI-derived λ_∥_ decreased by 7% (*p* = 0.11) in the PBS cohort, while no change was seen in the Dex-treated cohort. DBSI-derived λ_⊥_ increased by 41% (*p* < 0.05) and 16% (*p* = 0.24) in PBS- and Dex-treated EAE mice, respectively, at the end of 2-week treatment. At 1 month after treatment, moderate but not statistically significant DBSI λ_∥_ decrease was observed in both PBS- and dexamethasone-treated optic nerves comparing to their baseline (decreased by 4% and 2% respectively, **B**). Significantly elevated DBSI λ_⊥_ was seen at 1 month after PBS treatment (increased by 59%, *p* < 0.05, **C**) but not statistically significant in dexamethasone-treated optic nerves (increased by 20%, *p* = 0.14, **C**) comparing to their baseline. Two months after treatment, DBSI λ_⊥_ increase was apparent at 2 months after ending PBS (increased by 27%, *p* = 0.28, **C**) and dexamethasone treatment (increased by 35%, *p* = 0.02, **C**) from baseline. There was no statistical difference between treatment groups at each single time point. *Indicates *p* < 0.05, comparing to baseline.

**FIGURE 4:**
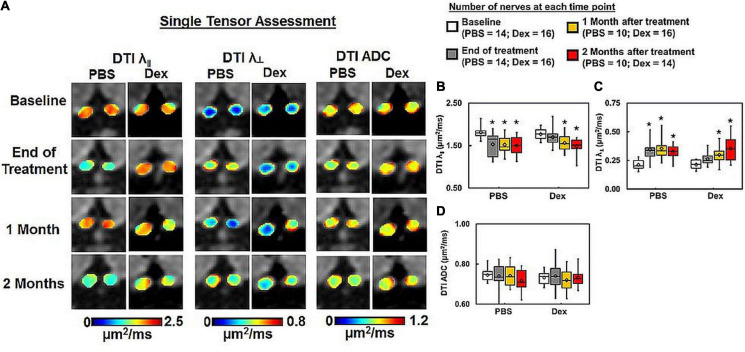
DTI-derived axial (λ_∥_) and radial (λ_⊥_) diffusivity, and ADC maps **(A)** from the same two representative mice as in Figure 2. PBS-treated optic nerves showed exaggerated DTI λ_∥_ decrease **(B)** and DTI λ_⊥_ increase **(C)** than DBSI λ_∥_ (Figure 3B) and DBSI λ_⊥_ (Figure 3C). DTI-derived ADC showed no difference within or between groups **(D)**. * indicates *p* < 0.05, comparing to baseline.

At the end of 2-week treatments, DBSI λ_| |_ (putative marker of axonal injury) of Dex-treated optic nerves was not decreased compared with the baseline value (*p* = 0.96, Table 1). DBSI λ_| |_ of PBS-treated optic nerves moderately decreased by 13% from the baseline value although not reaching statistical significance (*p* = 0.11, Figure 3B and Table 1). Compared to the baseline, DBSI λ_⊥_ (putative marker of myelination) in PBS-treated optic nerves increased by 41% (*p* = 0.01, Table 1) while Dex-treated DBSI λ_⊥_ increased non-significantly by 16% (Figure 3C and Table 1). A moderate but not significant DBSI λ_||_ decrease was observed in both PBS- and Dex-treated optic nerves at 1 month (decreased by 4% and 2% respectively, Table 1) and 2 months (decreased by 8% and 6% respectively, Table 1) after treatment (Figure 3B). Increased DBSI λ_⊥_ was seen at 1 month after PBS treatment (increased by 59% from baseline, *p* = 0.006, Figure 3C and Table 1) but was not significantly increased in Dex-treated optic nerves (increased by 20% from baseline, *p* = 0.15, Figure 3C and Table 1). A moderate but not significant DBSI λ_⊥_ increase by 27% from baseline was seen at 2 months after PBS (*p* = 0.27, Table 1). In contrast, the Dex-treated group had 35% DBSI λ_⊥_ increase from baseline (*p* = 0.02, Figure 3C and Table 1). However, DTI λ_| |_ (Figures 4A,B and Table 1) and DBSI λ_⊥_ (Figures 4A,C and Table 1) results were exaggerated and consistent with the change of DBSI hindered (Figure 2B) and restricted (Figure 2C) fraction, suggesting DTI result might be contaminated inflammatory pathology. In addition, DTI ADC (Figure 4D and Table 1) could not reflect damage in either PBS- or Dex-treated optic nerves.

### Dexamethasone Treatment Failed to Prevent Axonal Loss

**FIGURE 5:**
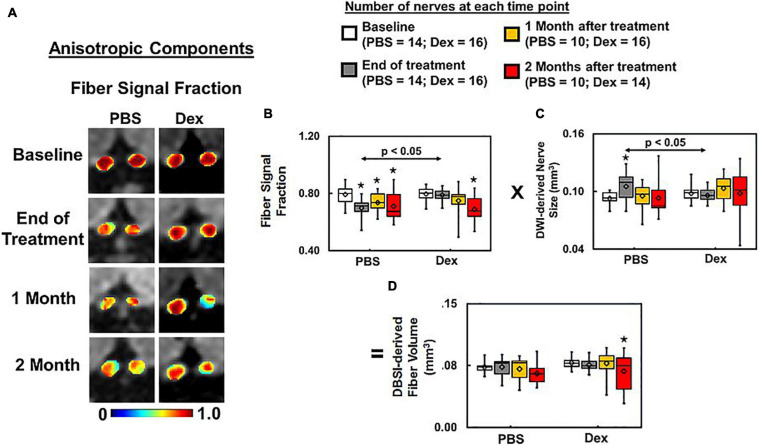
DBSI fiber signal fraction is the portion of the total diffusion signal within an image voxel that is putative biomarker of axonal density **(A)**. Decreased axonal density from baseline was apparent at all time-points (*p* < 0.05) after ON onset in the PBS treated mice, and at 1 and 2 months after Dex (*p* < 0.05) treatment (**A**, same representative mice of Figures 2, 3). Dexamethasone treatment effectively maintained the baseline fiber signal fraction at 2 weeks after treatment, slightly decreased by ∼10% from baseline without reaching statistical significance at 1 and 2 months afterward (**B**, *p* = 0.37). The DBSI “fiber volume” **(D)** was derived as DBSI anisotropic diffusion fiber signal fraction **(B)** multiplied by DWI-derived nerve volume **(C)** to reflect total fiber signal amount without dilution effects from cell inflammation/edema, putatively estimating the extent of axon loss **(D)**. DBSI-derived fiber volume quantifies the severity of axonal loss although not a true volume. At 2 months after treatment, axonal loss was seen in both PBS- and dexamethasone-treated optic nerves, suggesting that dexamethasone was not able to prevent irreversible axonal degeneration. * indicates *p* < 0.05, comparing to baseline.

Optic nerve DBSI fiber fraction (putative marker of apparent axon density) was decreased in PBS-treated but not Dex-treated mice at the end of the 2-week treatment (decreased from baseline by 12%, *p* = 0.0008 vs. 1%, *p* = 0.37 respectively, Figures 5A,B and Table 1). Significant optic nerve volume increase was detected in the PBS-treated optic nerves (increased by 13% from baseline, *p* = 0.0147, Figure 5C and Table 1) at the end of the 2-week treatment. In contrast, there was no detectable change in nerve volume at any measured time point in Dex-treated optic nerves (decreased by 2% from baseline, *p* = 0.6232, Figure 5C and Table 1) at the end of 2-week treatment. Comparing to Dex-treated group, significant lower fiber signal fraction was observed at the end of 2-week treatment (*p* = 0.0381, Figure 5B and Table 1). Meanwhile, significant increased nerve volume was detected at the end of 2-week treatment (*p* = 0.0012, Figure 5C and Table 1). Significantly lower fiber signal fraction was seen in both PBS- and Dex-treated optic nerves at 2 months after treatment (decreased by 10%, *p* = 0.0005 and 13%, *p* < 0.0001 from baseline respectively, Figure 5B and Table 1). There was no difference of fiber fraction (*p* = 0.858, Figure 5B and Table 1), nerve volume (*p* = 0.7252, Figure 5C and Table 1), and DBSI-derived fiber volume (*p* = 0.7096, Figure 5D and Table 1) between PBS- and Dex-treated groups at 2 months after treatment. These results suggest that Dex treatment offers no long-term benefits related to axon preservation.

### Immunohistochemistry (IHC) Staining of Optic Nerve

**FIGURE 6:**
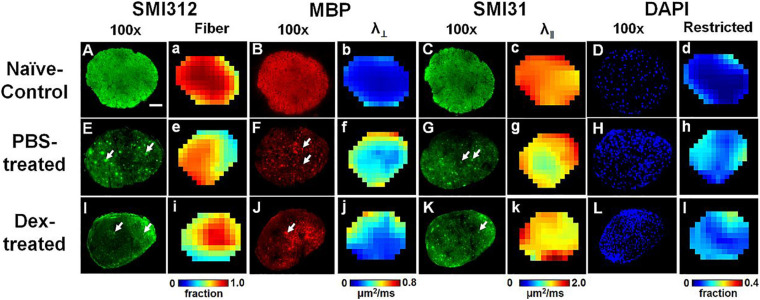
Representative 100× immunohistochemical staining images of total neurofilament (SMI-312, staining both injured and intact axons, **A,E,I**), myelin basic protein (MBP, assessing myelin integrity, **B,F,J**), phosphorylated neurofilament (SMI-31, reflecting intact axons, **C,G,K**), and 4′, 6-dianidino-2-phenylindole (DAPI, detecting nuclei, **D,H,L**) from naïve control (row 1), PBS-treated (row 2), and dexamethasone-treated (row 3) optic nerves at end of DBSI time course (2 months after treatment). The corresponding DBSI fiber signal fraction (the total signal of optic nerve tracts, **a,e,i**), radial diffusivity (λ_⊥_, myelin integrity of residual optic nerve tracts, **b,f,j**), axial diffusivity (λ_∥_, axonal integrity of residual optic nerve tracts, **c,g,k**) and restricted fraction (cellularity, **d,h,l**) metrics demonstrate injury patterns of optic nerve. Results here show different degrees of tissue damage in optic nerves from mice with optic neuritis treated with PBS versus dexamethasone. The results are consistent with *in vivo* DBSI findings that short-term treatment with dexamethasone does not protect optic nerves after the cessation of treatment. Optic nerves developed axonal injury (decreased staining intensity and swollen axons, the white arrows, in SMI-312, MBP and SMI-31 panels), demyelination (decreased staining intensity for MBP), axonal loss (decreased SMI-32 staining intensity and tissue shrinkage), and increased cellularity (increased DAPI staining). White scale bar: 50 μm.

**FIGURE 7:**
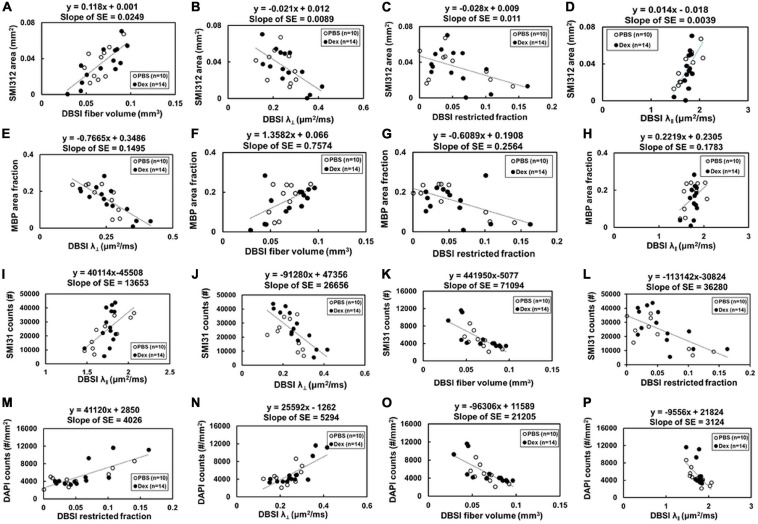
Optic nerve tissues were extracted and prepared for IHC staining after the last imaging time point (2 months post-treatment). The correlations of IHC and DBSI metrics were **(A–P)** were shown. The IHC biomarkers included SMI-312 area (absolute value of positive staining counts, **A–D**), MBP area fraction (the ratio of positive staining counts and total tissue area, **E–H**) and SMI-31 counts **(I–L)**, and DAPI counts **(M–P)** to reflect severity of axonal loss, demyelination, axonal injury, and cellularity, respectively. Total axonal counts as expected directly correlated with DBSI fiber volume **(A)** and DBSI-λ_| |_ **(D)**, inversely correlated with DBSI-λ_⊥_ (direct correlation with myelin integrity) and DBSI restricted diffusion fraction (increased inflammation impact axonal integrity). Similarly, MBP area (myelination integrity) directly corrected with DBSI axonal volume **(F)** and DBSI-λ_| |_ (**H**, i.e., axonal integrity impacted myelin integrity), and inversely correlated with DBSI-λ_⊥_ (**E**, increased myelination integrity) and DBSI-restricted fraction (**G**, increased inflammation also damaged myelin). Intact axonal staining (SMI-31) directly correlated with DBSI-λ_| |_ **(I)** and axonal volume **(L)**, i.e., DBSI axonal integrity markers, and inversely correlated with DBSI-λ_⊥_ (directly correlated with myelin integrity) and DBSI restricted fraction (**L**, inflammation damaged myelin integrity). Cellularity (DAPI staining) directly correlated with DBSI restricted fraction **(M)** and DBSI-λ_⊥_ (directly correlated with myelin damage), and inversely correlated with DBSI fiber volume **(O)** and DBSI-λ_| |_ (i.e., inversely correlated with axonal integrity). Results reflect that axonal pathologies in EAE optic nerves are inter-dependent due to the pathological dependence.

Comparing IHC of naïve optic nerve (Figures 6A–D), IHC of PBS-treated (Figures 6E–H) and Dex-treated (Figures 6I–L) optic nerves at end of the experiment showed decreased SMI-31 (intact phosphorylated axons) and SMI-312 (intact plus injured axons) staining intensity with irregular distribution of expanded hyper-intense areas due to axonal injury and axonal swelling (white arrows, Figures 6E,G,I,K), which were detected by DBSI fiber signal fraction (Figures 6a,e,i) and DBSI λ_*∥*_ (Figures 6c,g,k). Reduced MBP (myelin basic protein) staining intensity and irregular hyper-intense spots (white arrows, Figures 6F,J) resulting from demyelination and possible myelin debris was seen in both PBS- and Dex-treated optic nerves, and the results was consistent with DBSI λ_*⊥*_ (Figures 6b,f,j). Increased DAPI counts (number of cell nuclei) was also observed in PBS- and Dex-treated optic nerves (Figures 6H,L) and consistent with DBSI restricted fraction (Figures 6d,h,l). Decreased SMI-312 staining intensity in PBS- and Dex-treated optic nerves were associated with noticeable axonal loss (Figures 6E,I). SMI312 area, MBP fraction, SMI31 counts, and DAPI counts associated with DBSI-derived fiber volume (Figure 7A, directly correlated), DBSI λ_⊥_ (Figure 7E, inversely correlated), DBSI λ*_| |_* (Figure 7I, directly correlated), DBSI restricted isotropic fraction (Figure 7M, inversely correlated), suggesting that DBSI derived pathological metrics revealed the severity of axonal loss, demyelination, axonal injury, and cell infiltration. In this study, the change of DBSI λ_⊥_ was also associated with SMI312 area (Figure 7B, inversely correlated), SMI31 counts (Figure 7J, inversely correlated), and DAPI counts (Figure 7N, directly correlated). The change of DBSI-derived fiber volume was associated with SMI31 counts (Figure 7K, directly correlated) and DAPI counts (Figure 7O, inversely correlated). The change of DBSI λ*_| |_* was associated with MBP (Figure 7D, directly correlated). The change of DBSI restricted fraction was associated with SMI31 (Figure 7L, inversely correlated). Correlations among IHC and DBSI metrics indicate that optic nerve pathologies were inter-dependent reflecting the inter-dependence among inflammation, demyelination, and axonal injury in optic neuritis of EAE mice. IHC scatter plot distributions overlapped between treatment groups at 2 months, suggesting that the 2-week treatment dexamethasone treatment had little impact on long-term optic nerve pathologies. Overall, the data indicate that *in vivo* findings of DBSI metrics reflected underlying neuropathology.

## Discussion

Diffusion basis spectrum imaging has shown success in modeling non-Gaussian diffusion phenomena with multiple Gaussian functions for biological tissues and environment in MS subjects and EAE mice (Wang et al., 2011a, 2015; Chiang et al., 2014; Lin et al., 2017). In this study, we used DBSI to assess the effects of 2-week Dex treatment on optic nerve integrity in murine ON serially over the subsequent 2 months, with immunohistochemistry at the conclusion of study time course to assess optic nerve neuropathology. A two-sample Student’s *t*-test of DBSI restricted fraction (putative biomarker for inflammation) was used to estimate the sample size (*n* = 7) needed to achieve the statistical significance. During the experiment, five PBS-treated EAE mice died at various time points leading to the small sample size for this study. Despite the small cohort size, the longitudinal comparison within individual EAE mice demonstrated the difference between PBS- and Dex-treatments. It is much close to clinical need to design the personal treatment strategy, especially for people with MS (Gajofatto and Benedetti, 2015). Our findings were still consistent with the classic human ONTT trial (Gal et al., 2012), finding that a short course of corticosteroids led to improved visual function in the short-term while it failed to preserve visual function in the long-term (Figure 1B). Histologically, similar degrees of optic nerve axonal injury/loss were observed in both PBS- and Dex-treated mice at 2 months after the 2-week treatment. To the best of our knowledge, this is the first study to non-invasively and longitudinally examine the effects of corticosteroids on the evolution of optic nerve pathologies of ON mice.

Corticosteroids suppress inflammation through inhibiting vascular permeability, suppressing leukocyte emigration into sites of inflammation, and reducing production of inflammatory mediators (Perretti and Ahluwalia, 2000; Coutinho and Chapman, 2011). Although corticosteroids are commonly employed to treat acute inflammation, they have known associated adverse effects, some of which are cumulative (Ohno et al., 1987; Buchman, 2001; Myhr and Mellgren, 2009), limiting its long-term use. Our findings were consistent with ONTT conclusion that no long-tern benefits of steroids for VA improvement. We speculate that timing of treatment commencement may play a critical role in treatment efficacy. Thus, with accurate and non-invasive assessment of optic nerve pathologies using DBSI that is capable of detect subclinical pathologies could improve the treatment efficacy by affording an early treatment before clinical manifestations detectable in MS (Noyes and Weinstock-Guttman, 2013; Kavaliunas et al., 2017). In the current study, we started at 0.1–0.3 mg/kg dexamethasone (Donia et al., 2010) that resulted in inconsistent and limited effects on EAE mice. Our final working dose of dexamethasone for treating ON (10 × clinical dose) is comparable to that used in previous reports to treat optic neuritis of MOG_35__–__55_ EAE mice (Wust et al., 2008; Donia et al., 2010). The anti-inflammation effect of Dex seen in the report was also detected in the present study, manifested as the lower restricted isotropic diffusion fraction than PBS-treated EAE mice by comparing to baseline within group.

Axonal loss is believed to be the primary substrate of irreversible neurological disability in MS (van Waesberghe et al., 1999; Bjartmar et al., 2000; Wujek et al., 2002; Van Asseldonk et al., 2006). Optical coherence tomography (OCT) has been increasingly relied upon as a non-invasive biomarker of axonal loss for people with MS (Costello et al., 2006a; Lagreze et al., 2009; Saidha et al., 2015). DTI-derived fractional anisotropy (FA) has also been implied to reflect axonal injury. However, acute inflammation-associated cell infiltration and vasogenic edema might lead to optic nerve swelling. Both DTI and OCT results might be masked by these inflammations associated changes. Our results indicated that DTI λ_∥_ and λ_⊥_ was affected by the progression of inflammation overestimating axonal pathologies (Figures 2B,C). Thus, DTI metrics would fail to accurately reflect axonal injury or demyelination in the presence of axonal loss and/or inflammation. In contrast, DBSI not only detects inflammatory pathologies but also reflects axonal injury and demyelination without confounding effects of inflammation. DBSI-derived fiber volume, i.e., DBSI fiber fraction multiplied by optic nerve volume, quantified axonal loss of the optic nerve and spinal cord in the presence of acute inflammation-associated swelling (Lin et al., 2017, 2019). In the present study, DBSI-derived fiber volume reflected histology-detected axonal loss, and non-invasively reflected the failure of dexamethasone to prevent long-term optic nerve axonal loss in living mice.

In the current study, SMI312, MBP, SMI31, and DAPI were used to validate the specificity and sensitivity of DBSI-derived fiber volume, DBSI λ_⊥_, DBSI λ_∥_, and DBSI restricted fraction. However, the inter-dependence among IHC biomarkers and DBSI metrics seen in the current study (Figure 7) reflects the potential causal relationships among underlying pathologies of optic neuritis in EAE mice. The results imply that DBSI metrics may not uniquely correlated with the target pathologies since one cannot definitively isolated inter-relationship between metrics. This observation is likely to hold true for all MRI derived biomarkers that are derived based on morphological changes without molecular specificity. For complex pathologies in diseases such as multiple sclerosis, it would be difficult to definitively validate any pathological biomarkers since the underlying pathologies are inter-dependent.

Histological validation of *in vivo* MRI findings needs to take into account of the evolution of pathologies of MS/EAE to elucidate the potential inter-correlations among coexisting individual pathological components. For example, if at a lesion or normal appearing white matter site where inflammation induces subsequence axonal injury at the same site or in close vicinity, then inflammation and axonal injury would correlate with each other. Under this scenario, an inflammatory marker could correlate with axonal injury or vice versa. Our previous numerous studies on EAE mice and postmortem MS specimens favorably suggest that DBSI-derived pathological metrics are adequate biomarkers of pathologies of axon, myelin, and inflammation origin. However, due to the unspecific nature of MRI biomarkers of white matter injury it would require researchers to be cautious in applying these markers in complex pathologies present in MS/EAE.

In summary, we employed serial DBSI to assess optic nerve pathology longitudinally in living EAE mice. Optic nerve responses to dexamethasone and PBS treatments, showing short-term but not long-term benefits of corticosteroids, which recapitulated observations from the ONTT in ON patients. Upon comparing *in vivo* DBSI to neuropathology, we demonstrated that DBSI-derived fiber volume can serve as a quantitative biomarker of axonal loss. Measurement of axonal loss is important, as it underpins permanent neurological impairment. The current study provides an important validation of DBSI-derived pathological markers in response to a treatment, and uniquely quantifies axonal loss *in vivo*.

## Data Availability Statement

The raw data supporting the conclusions of this article will be made available by the authors, without undue reservation.

## Ethics Statement

The animal study was reviewed and approved by Washington University Institutional Animal Care and Use Committee (IACUC) and conformed to the NIH Policy on Responsibility for Care and Use of Animals.

## Author Contributions

T-HL, AC, and S-KS contributed to the concept and experimental design. T-HL, PS, and CS contributed to the protocol and code development. T-HL, JZ, CS, MW, XN, RY, and S-KS contributed to the generation, collection, and analysis of data. T-HL, AC, RY, MW, and S-KS contributed to the manuscript drafting. AC and S-KS contributed to the critical review of the manuscript. T-HL, JZ, CS, MW, PS, XN, RY, AC, and S-KS contributed to the manuscript approval. All authors contributed to the article and approved the submitted version.

## Conflict of Interest

The authors declare that the research was conducted in the absence of any commercial or financial relationships that could be construed as a potential conflict of interest.

## Footnotes

1 https://imagej.nih.gov/ij/
